# Feasibility of Synchrotron-Based Ultra-High Dose Rate (UHDR) Proton Irradiation with Pencil Beam Scanning for FLASH Research

**DOI:** 10.3390/cancers16010221

**Published:** 2024-01-03

**Authors:** Lingshu Yin, Umezawa Masumi, Kan Ota, Daniel M. Sforza, Devin Miles, Mohammad Rezaee, John W. Wong, Xun Jia, Heng Li

**Affiliations:** 1 Department of Radiation Oncology and Molecular Radiation Sciences, Johns Hopkins University School of Medicine, Baltimore, MD 21205, USA; dsforza1@jhmi.edu (D.M.S.); dmiles18@jhmi.edu (D.M.); mrezaee1@jhmi.edu (M.R.); jwong35@jhmi.edu (J.W.W.); xunjia@jhu.edu (X.J.); hengli@jhu.edu (H.L.); 2 Hitachi, Ltd., Research and Development Group, Center for Technology Innovation–Energy, 7-2-1, Omika-chou, Hitachi-shi 319-1292, Ibaraki-ken, Japan; masumi.umezawa.fj@hitachi.com; 3 Pyramid Technical Consultants, Inc., Boston, MA 02452, USA; kota@ptcusa.com

**Keywords:** proton, synchrotron, FLASH, ultra-high dose rate, pencil beam scanning

## Abstract

**Simple Summary:**

Ultra-high dose rate (UHDR) irradiation in proton therapy has mostly been performed using a cyclotron system so far. In this study, we present our approach to achieve an ultra-high dose rate for a clinical synchrotron proton therapy system and its dosimetric specifications. We demonstrated that it is feasible to adapt an existing clinical synchrotron-based proton therapy system and reach ~100 nA nozzle beam. UHDR dose rate (>40 Gy/s) was reached with the tested spot scanning patterns. The maximum dose per spill depends on the field size and is limited by the maximum charge extraction per spill. A beam monitoring ion chamber is currently being incorporated into the beam control system to improve system reproducibility and expand the irradiation area.

**Abstract:**

Background: This study aims to present the feasibility of developing a synchrotron-based proton ultra-high dose rate (UHDR) pencil beam scanning (PBS) system. Methods: The RF extraction power in the synchrotron system was increased to generate 142.4 MeV pulsed proton beams for UHDR irradiation at ~100 nA beam current. The charge per spill was measured using a Faraday cup. The spill length and microscopic time structure of each spill was measured with a 2D strip transmission ion chamber. The measured UHDR beam fluence was used to derive the spot dwell time for pencil beam scanning. Absolute dose distributions at various depths and spot spacings were measured using Gafchromic films in a solid-water phantom. Results: For proton UHDR beams at 142.4 MeV, the maximum charge per spill is 4.96 ± 0.10 nC with a maximum spill length of 50 ms. This translates to an average beam current of approximately 100 nA during each spill. Using a 2 × 2 spot delivery pattern, the delivered dose per spill at 5 cm and 13.5 cm depth is 36.3 Gy (726.3 Gy/s) and 56.2 Gy (1124.0 Gy/s), respectively. Conclusions: The synchrotron-based proton therapy system has the capability to deliver pulsed proton UHDR PBS beams. The maximum deliverable dose and field size per pulse are limited by the spill length and extraction charge.

## 1. Introduction

When compared to irradiation with conventional dose rate (CONV), ultra-high dose rate (UHDR) radiation (>40 Gy/s) has demonstrated improved normal tissue sparing and equivalent tumor control, referred to as the “FLASH effect” [1]. This FLASH effect has been reported in a multitude of pre-clinical studies with different animal models across a variety of treatment sites [2,3,4,5,6,7,8,9,10,11,12,13,14,15]. With the encouraging results from these studies, FLASH radiation therapy has swiftly moved forward, with several clinical trials open for canine [16] and human cancer patients [17].

The delivery modalities for UHDR irradiation include X-rays from orthovoltage units [2] or experimental super-conducting LINAC [2,3,4,5,6,7,8,9,10,11,12,13,14,15], electron beams from modified clinical LINAC [2,3,4,5,6,7,8,9,10,11,12,13,14,15] and proton therapy systems [12,13,14,15]. Among these modalities, UHDR proton therapy has gained particular interest, stemming from its desirable beam properties such as a capability to treat deep tumors, sharp beam penumbra and distal dose fall-off (Bragg peaks). Most of the reported proton UHDR studies have utilized clinical isochronous cyclotron [12,13,14,15,18,19,20,21] or synchro-cyclotron systems [22,23], albeit with necessary modification to increase the proton beam current and attain the desired dose rate. These cyclotron systems deliver quasi-continuous proton beams with the beam current typically on the order of hundreds of nano-amperes (nA). In contrast, the synchrotron-based proton therapy system delivers proton beams in sequential pulses known as synchrotron spill, each lasting 0.5~5 s for double scattering beams or up to a few seconds in the slow extraction mode for PBS. The total charge of protons per spill varies from ~3 to 15 nC, and an approximately 2 to 3 s delay between spills is necessary to re-fill the synchrotron ring with protons and for further acceleration. The average beam current remains at a few nAs, while the total number of protons per spill limits the maximum dose that can be delivered in each pulse. 

Due to these challenges, few FLASH studies have been reported using synchrotron-based proton therapy systems. The total number of extracted protons per spill and the delay time between spills are inherently limited by the design of synchrotron. Due to the space-charge limit and slow dipole cycle rate in clinical synchrotron systems, there is no significant difference in the charge extraction per spill between UHDR and clinical mode. The most feasible approach for synchrotron-based proton UHDR irradiation is to increase the power of the beam extraction RF, shortening each spill to the order of milliseconds. This approach, as demonstrated at other synchrotron facilities [24,25,26], would effectively increase the beam current to ~100 nAs. If entire proton irradiation can be delivered in a single spill, the field-averaged dose rate could potentially reach >40 Gy/s under certain scenarios, therefore meeting the requirements for UHDR irradiation. Notably, a synchrotron-based proton UHDR study has been reported recently by Yang et al. [24]. Their approach, with passive scattering techniques, requires significant hardware modifications to the beamline using meticulously designed scatterer to increase the field size and ridge filter for energy modulation [25]. The limited efficiency of beam transport in passive scattering proton UHDR beams imposes constraints on its potential applicability within other synchrotron systems. Meanwhile, PBS has become the standard method for treatment delivery in modern proton therapy systems. Cyclotron-based proton UHDR irradiation with PBS has been used for pre-clinical studies. While there are certain challenges, the remarkable versatility of PBS in fluence modulation, especially when combined with ridge filters for energy modulation, has emerged as the central focus in the treatment planning studies for proton FLASH therapy.

In this study, we describe our approach to implement UHDR PBS capability on a synchrotron-based proton therapy system. Our work demonstrates that such an adaptation is feasible without the hurdles of significant changes to the beamline hardware. While we encountered certain challenges and limitations inherent in the current-generation synchrotron proton therapy system, the proton UHDR beamline developed at our institution offers opportunities for in-depth investigation into the fundamental mechanisms of FLASH sparing effects and facilitates preclinical small animal proton UHDR irradiation experiments.

## 2. Materials and Methods

### 2.1. Modification to Existing Beam Delivery and Control System

The proton therapy system used in this study (Hitachi Probeat CR, Hitachi Ltd., Tokyo, Japan) is a multi-room synchrotron system specifically designed for proton PBS with smaller gantry footprint. The fixed-beam treatment room in this system was fitted to a dedicated research room for pre-clinical studies and FLASH experiments.

The synchrotron system delivers proton beams on a spill-by-spill basis. In each spill, a limited number of protons can be accelerated to 7 MeV by the LINAC and injected into the synchrotron acceleration ring. A single injected punch of protons is accelerated to a user-specified energy roughly between 70.2 and 228.7 MeV and slowly extracted for irradiation at a conventional dose rate. In the UHDR mode, beam extraction RF patterns were adjusted for irradiation at 142.4 MeV and 228.7 MeV by the Hitachi engineering team. These RF patterns were experimentally optimized to both maximize the number of protons that can be extracted for each spill and minimize beam output fluctuations.

The first challenge in this study is to effectively control the delivery of proton UHDR beams. Within this dedicated PBS system, the beam monitoring ion chamber (BMIC) located in the PBS nozzle is the sole ion-chamber available for monitoring beam output and regulating beam delivery. During UHDR irradiation, the BMIC must be disconnected and cannot be used for beam delivery control. Instead, we utilized the simulated pulse signal interface (SPSI) in the Hitachi synchrotron system for beam control. The square wave pulse signal from a waveform generator (Keysight 33500B, Keysight Technologies, Santa Rosa, CA, USA) was supplied to the beam control system through SPSI. The system is configured to terminate the synchrotron spill after 500 counts of square pulses have been received. Therefore, the spill length can be adjusted by the frequency of the pulsed signal to SPSI. For example, a 50 ms proton spill can be requested by setting the pulse frequency to 10 kHz. Adjusting the frequency to 12.5 kHz will change the spill length to 40 ms, etc. Without the actual signal from BMIC, each synchrotron spill is terminated at a preset time regardless of the number of protons that have been extracted. Therefore, this leads to issues regarding the reproducibility of beam output, as well as challenges in implementing PBS for UHDR irradiation; both will be discussed later. 

The other challenge in this study is to achieve an acceptable field size to irradiate radio-biological samples. The preclinical studies proposed for this UHDR beamline involve a sample size at 5 × 5 × 1 mm^3^ and a dose at Bragg peak > 120 Gy per spill. However, the maximum total charge per spill (~5 nC) is relatively small. Retro-fitting this beamline with beam scatterer and collimators would increase the field size at the cost of reduced transport efficiency, leading to a lower deliverable dose. Therefore, we opted to utilize the existing pencil beam scanning capability in this beamline to achieve the desired field size. In UHDR mode, pencil beam scanning can be performed using the scanning controller in the same manner as in the clinical mode. Since the BMIC signal is replaced by the fixed frequency pulse signal through SPSI, the spot MU is proportional to the spot dwell time rather than the actual number of protons in each spot. The measured beam fluence rate can be used to derive the dwell time for individual spots, such that each spot will deliver an approximately equal number of protons. Therefore, the irradiation field size can be effectively increased from single spot to a scanning field size up to a few millimeters. 

### 2.2. Pulse Structure Measurement of the Synchrotron Spill in UHDR Mode

The total charge of extracted protons per spill was measured using a Faraday cup, which was installed on the beamline between the exit of the accelerator ring and the beam entrance to the fixed beam room.

**Figure 1:**
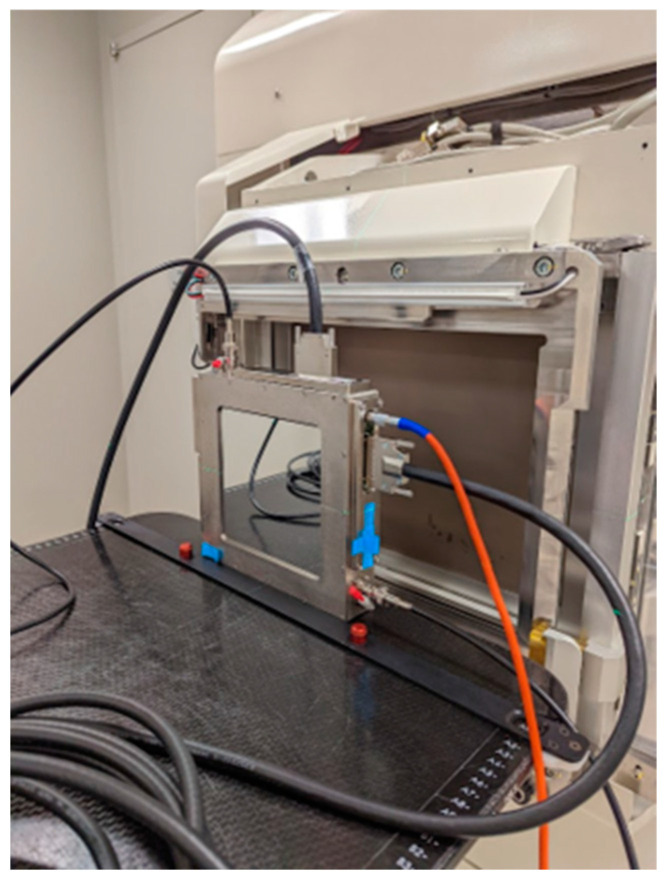
Setup of IC64-6 in front of the nozzle for pulse structure measurement.

To determine the time structure of proton pulses, including spill length, pulse frequency and microscopic pulse structure, a transmission strip ion chamber (Figure 1, IC64-6, Pyramid Technical Consultants, Waltham, MA, USA) and a high-speed 128-channel charge integrator (I128, Pyramid Technical Consultants, Waltham, MA, USA) were employed. The transmission ion chamber was designed with two sets of 64 parallel strip electrodes at a 1 mm pitch in both the X and Y directions to accurately record the position and profile of the proton beam. Additionally, it features an integral dose plane with 1 mm gap and 1000 V bias voltage to capture the entire beam output. The chamber’s performance under proton UHDR irradiation, including linearity, charge collection efficiency, and other characteristics, has been thoroughly characterized using proton beams from a cyclotron system with over 300 nA beam currents [27]. The sampling frequency of the charge integrator was set at 10 kHz, sufficient for the measurement of the microscopic pulse structure within each synchrotron spill.

### 2.3. Measurement of Beam Characteristics in UHDR Mode

We do not expect the beam characteristics (energy, spot size and spot position, as well as beam divergence) to be significantly different between the clinical and UHDR mode. Nevertheless, in UHDR mode, beam optics may exhibit slight variations owing to the space charge effect caused by densely bunched protons. Beam tuning is also likely to be adjusted due to the implementation of new extraction RF patterns. Most critically, certain safety interlocks, including feedback mechanisms for spot size and position from BMIC, are temporarily bypassed with SPSI in UHDR mode. As a precautionary measure, we decided to measure some aspects of the beam characteristics and confirm its consistency between UHDR and clinical mode.

Gafchromic^TM^ film (Ashland Inc., Bridgewater, NJ, USA) has been widely used for dosimetry purposes in UHDR studies. The dose response of Gafchromic^TM^ film was found to be consistent with dose rates up to 1500 Gy/s in previous studies [28,29,30]. Specifically, the MD-V3 film has a large dynamic dose range (>100 Gy), making it suitable for the cross-comparison of proton beam dosimetry at conventional and UHDR dose rates. It was used extensively in this study to quantify the characteristics of proton UHDR beams such as the spot size and spot position, as well as absolute dose profile, in proton UHDR irradiations. 

The spot size and spot positions were measured with MD-V3 film in both clinical and UHDR mode using a spot pattern containing nine spots (4 spots with 10 cm grid spacing, 4 spots with 5 cm grid spacing and 1 spot at the central axis). As a standard commissioning requirement to derive beam model parameters associated with the propagation of in-air spot size and beam divergence, the measurement of this spot pattern was repeated at several positions along the beam axis. These positions ranged from 40 cm upstream to 20 cm downstream of the isocenter, with intervals of 10 cm.

### 2.4. Measurement of Dose and Dose Profile in UHDR Mode

For the preliminary dosimetry measurement, we used a 2 × 2 rectangular spot pattern with various spacings to create square fields at different depths (2, 5, 10 and 13.5 cm depth) in a Solid Water^TM^ HE phantom (Sun Nuclear, Melbourne, FL, USA). Each measurement was repeated five times using MD-V3 films to measure the absolute dose and dose profile at each depth. To account for the response dependence on proton linear energy transfer (LET), calibration for film dosimetry was conducted at each depth of measurement, respectively, in clinical mode, covering a range of doses from 5 Gy to 140 Gy. These films were scanned with an Epson Expression 12000XL scanner (Epson, Nagano, Japan) with 0.05 mm pixel resolution (508 dpi). Film calibration curves were created following the established protocol and applied to those irradiated in UHDR mode [31].

## 3. Results

### 3.1. Pulse Structure and Beam Fluence

**Figure 2:**
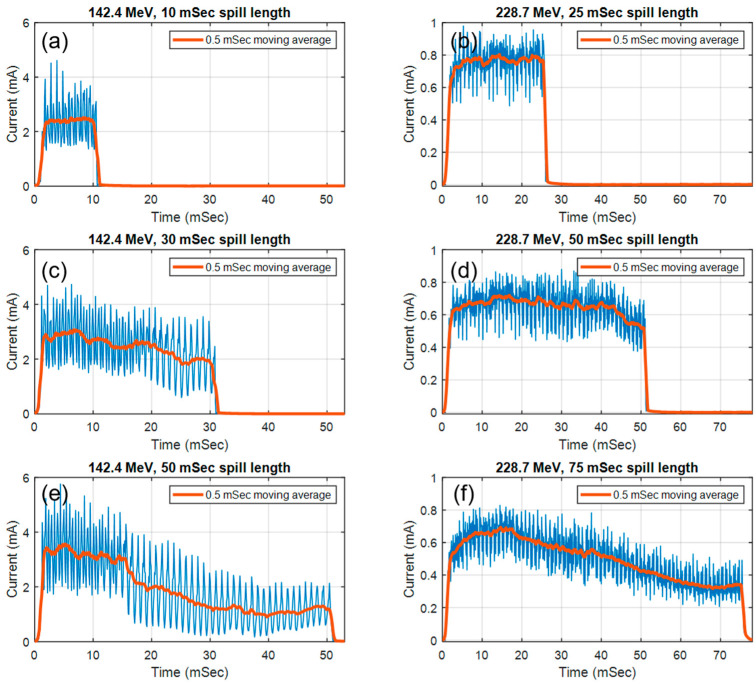
The pulse structure of proton UHDR spills at 142.4 MeV (**a**,**c**,**e**) and 228.7 MeV (**b**,**d**,**f**). The sampling interval of the integral dose plane in IC64-6 was set to 0.1 ms. The raw signal (blue line) was smoothed with a moving average of 0.5 ms (red line) to remove high frequency noise.

After preliminary tests with the beamline and extraction RF parameters, the maximum spill length was determined at 50 ms for 142.4 MeV and 75 ms for 228.7 MeV beams, respectively. Each spill is separated by a two second delay to inject and accelerate protons in the synchrotron. The microscopic pulse structure of synchrotron spills in UHDR mode is shown in Figure 2. At both energies, due to the design of the RF pattern, the beam current will reach its peak at the beginning of each spill to maximize the number of protons that can be extracted and will gradually decrease until the end of the spill. At 142.4 MeV, the current measured in the integral dose plane of IC64-6 is approximately 3.4 mA at the beginning of the spill and gradually decreased to ~1.3 mA (62% lower) at the end of the 50 ms spill. 

The extracted charge with different spill lengths were summarized in Table 1 and Table 2. The maximum number of protons that can be extracted per spill is 4.96 ± 0.10 nC (at 142.4 MeV) and 3.08 ± 0.28 nC (at 228.7 MeV), respectively. This leads to average beam currents at 99.20 nA and 41.07 nA at each energy level.

As previously mentioned, due to the “peaked” RF design, shorter spills will generate a higher beam current and larger output fluctuations. Longer spills will produce a more stable output at the cost of a lower beam current. At the shortest spill length tested in our study (10 ms at 142.4 Mev and 9 ms at 228.7 MeV), the equivalent beam current increased to 143.20 nA and 53.44 nA; however, the standard deviation of the Faraday cup readings also increased from 2.0% and 9.10% to 9.8% and 14.2%, respectively. Due to the internal averaging of the Faraday cup readings in the synchrotron controlling software, the standard deviation of the actual beam output is expected to be much larger.

### 3.2. Beam Characteristics

After considering the reproducibility of the beam and the maximum charge per spill as well as the practical feasibility for pre-clinical experiments, we selected 142.4 MeV for the UHDR experiments. Unless stated otherwise, the discussions and reported data in the following sections pertain exclusively to the UHDR or CONV measurements at 142.4 MeV. All the UHDR measurements in the remainder of this section were conducted with 50 ms spills at 142.4 MeV. 

**Figure 3:**
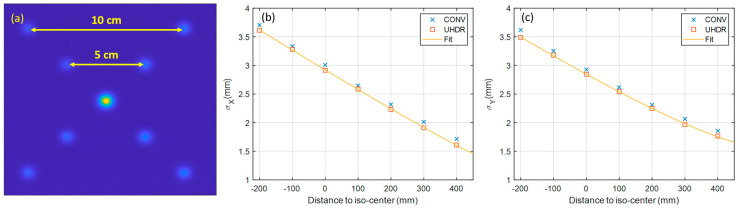
Measured spot patterns at iso-center (**a**) and spot sizes along the CAX (**b**,**c**).

The measured spot size and its propagation along CAX are shown in Figure 3. The spot sigma at both the X and Y axis at UHDR dose rate are within 0.1 mm from the measurements at the conventional dose rate. The spot position error was found to be 0.23 ± 0.19 mm.

**Figure 4:**
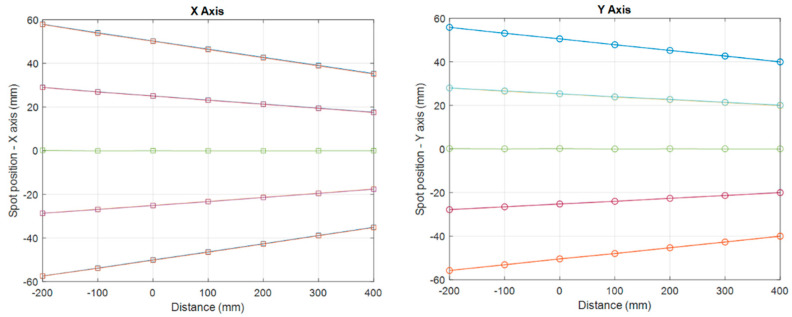
Measured spot position at different positions along the CAX.

The measured spot position and its propagation along the CAX are shown in Figure 4. Each line represents the trajectory of individual proton spots on the X or Y axis, whereas the intersection of lines represents the position of the virtual source. The virtual source to axis distance (SAD) was found to be 1342.41 ± 12.98 mm and 1924.79 ± 16.39 mm for X and Y axis, respectively, within 6 mm from the virtual SAD at conventional dose rate (1342.36 mm and 1919.10 mm, respectively). These differences in the spot size and virtual SAD are considered negligible, and hence the consistency of beam characteristics between UHDR and CONV dose rates is confirmed.

**Figure 5:**
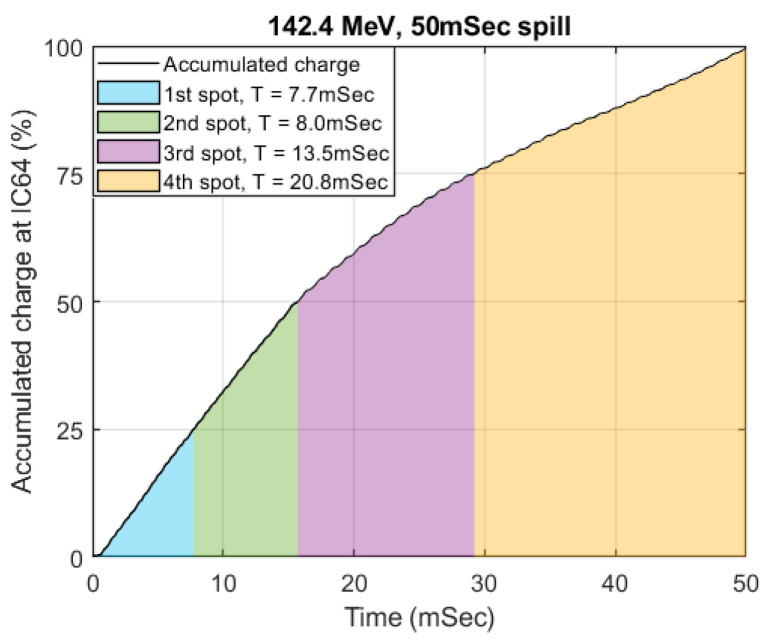
Accumulated charge collected at IC-64 and corresponding spot dwell time for four equally weighted spots.

The beam fluence measured at the integral dose plane in IC64-6 was used to derive the spot dwell time for 50 ms spill at 142.4 MeV. The cumulative sum of the curve in Figure 2e was plotted in Figure 5. The spot dwell time can be derived by interpolating the curve in Figure 5 according to the desired spot weight on the *Y*-axis. For a 2 × 2 spot delivery pattern, we estimated that the spot dwell time should be 7.7, 8.0, 13.5, 20.8 ms, respectively, such that each spot will deliver approximately the same number of protons. Since the beam current is at its highest at the beginning of the spill and gradually decreases towards the end of the spill, the dwell time of each spot will sequentially increase to compensate for the decreasing beam current.

### 3.3. Film Dosimetry Results

**Figure 6:**
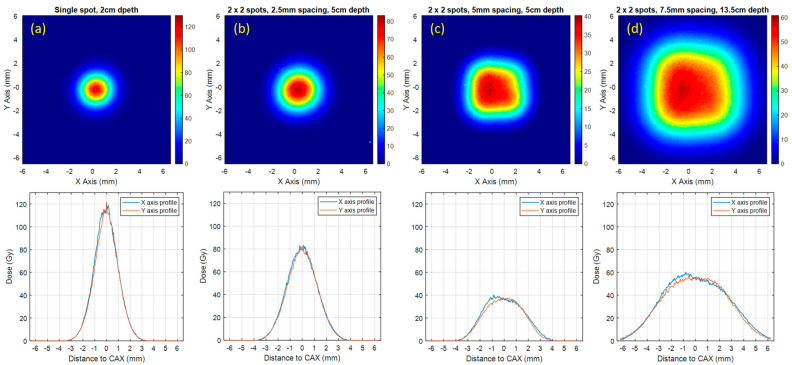
The 2D dose distribution (first row) and dose profile (second row) measured by MD-V3 films at various depth in the solid water phantom.

Based on the spot dwell time derived from Figure 5, MD-V3 films were used to measure the dose delivered per spill and dose profile for the 2 × 2 spot delivery patterns at various depths in the solid water phantom. During the measurement, the phantom surface was maintained at 30 cm upstream. The measurement depths were set to 2 cm, 5 cm and 13.5 cm. The dose distributions measured with MD-V3 films are shown in Figure 6. Due to the fluctuations of the beam current, the derived spot dwell time from Figure 5 does not always generate an ideal homogeneous dose distribution. For the UHDR spills shown in Figure 6c,d, it appears that the beam current was over-estimated at the last two spots, causing skewed dose profiles.

The summary of UHDR-related dosimetric indices such as dose per spill, dose rate and field size are shown in Table 3. The standard deviation of beam output is 4.6%, whereas the field size at D50% ranges from 2.1 mm to 6.9 mm with different spot patterns and depths. The average dose rate ranges from 726.3 Gy/s to 2436.2 Gy/s. In this study, we only tested spot patterns with 2 × 2 spots and different spot spacings. Theoretically, we can deliver more spots and expand the field size. The technical challenges and dosimetric impact of expanding the scanning field size will be discussed in the next section.

## 4. Discussion

Three key factors need to be considered when developing a synchrotron-based proton UHDR beamline: (1) the beam current within each spill, (2) total extraction charges per spill, and (3) the time delay between each spill. As demonstrated here and in previous studies, the extraction RF can be increased to generate a beam current sufficient for UHDR irradiation without much technical difficulty. We reported here, with this approach, that the beam current can reach ~100 nA at 142.4 MeV. The average dose rate of a single proton pencil beam spot can be higher than 2000 Gy/s at 100 nA beam current and 50 ms spill length.

However, due to the limited number of protons per spill and the delay time between each spill, it is technically challenging to irradiate a large volume with a UHDR dose rate within one spill. We reported that a maximum ~5 nC of protons can be extracted per spill at 142.4 MeV in our UHDR beamline. The maximum deliverable dose per spill falls off dramatically as the scanning field size increases. For the UHDR irradiation of large animals, the maximum charge per spill needs to be increased to accommodate larger target volumes. Although this would require modifications to the beamline, it is still feasible and not difficult. It is worth noting that the UHDR beamline retrofitted from the double scattering system has already been reported to be capable of delivering up to 16 nC per spill [25].

The development of the proton UHDR beamline at our institution currently faces limitations due to the absence of BMIC-based beam control. The estimation of beam output relied on the average number of protons per spill, while the assignment of individual spot weights was determined based on the measured proton fluence rate within a spill. Both aspects are susceptible to fluctuations in the beam extraction RF from spill to spill, resulting in a 4.6% variance in beam output and non-uniform fluence distribution within the scanning fields.

The absence of BMIC also constrains our ability to utilize PBS to expand the field size. The configuration of SPSI requires a minimum of 13 pulses from the external signal to generate a single pencil beam spot. Given a 10 kHz pulse signal and 500 pulses for a 50 ms spill, this criterion limits us to a maximum of 13 spots deliverable in each spill. Consequently, we have opted to restrict the spot patterns to a 2 × 2 configuration for the purposes of this feasibility study. This constrained delivery pattern keeps the scanning field size relatively small. Even with the most conservative dose rate definition, known as the “field dose rate” [32], the system still exhibits the capability to irradiate an area of 2.3 × 2.3 mm^2^, delivering 36.3 Gy per spill and maintaining a dose rate of 726.3 Gy/s at a depth of 5 cm in the plateau region. This dose rate significantly surpasses the established FLASH dose rate threshold of 40 Gy/s. Should we choose to segment the spill into more spots and expand the scanning field size to span a few centimeters, the dose delivered per spill would decrease due to the limited charge available per spill, and hence the average dose rate would be smaller as well. Nevertheless, the dose rate will likely meet the requirements for FLASH dose rates under certain field size limitations. Achieving this capability necessitates the implementation of a well-designed beam delivery control system based on BMIC for proton UHDR PBS irradiation.

The transmission ion chamber IC64-6 utilized in our study was designed for UHDR applications. It can generate pulse signals based on the accumulated charge from the integral dose plane. In theory, it can be used to control the beam and spot delivery properly through SPSI. We are currently working on the comprehensive characterization of this transmission ion-chamber and potential integration with the beam delivery system. The proper configuration of the charge quantum per pulse is crucial for the transmission ion chamber in the context of UHDR irradiation. In this study, we used a 10 kHz square wave signal for UHDR beam control. We have estimated that, to achieve the same precision as in clinical mode, the pulse rate from the ion chamber must be elevated by a factor of 100, reaching approximately 1 MHz at the peak beam current for delivering UHDR scanning beams. Furthermore, it is imperative to thoroughly investigate the charge collection efficiency, particularly in recombination effects within the BMIC under UHDR irradiations. Previous reports have indicated that a saturated nozzle BMIC operating at beam currents exceeding 30 nA can induce a roughly 5% beam-to-beam output variation and up to 10% daily fluctuations [20]. While the IC64-6 transmission chamber used in this study has been evaluated for charge collection efficiency and ion recombination effects using scattered proton beams at current up to 350 nA, it is essential to note that the smaller spot size in our system (σ = 2.0 mm versus 5.5 mm in a previous study [27]) may lead to higher ionization density and more likelihood of ion recombination. As such, this demands further in-depth investigation.

In this study, we predominantly used Gafchromic films for dosimetry measurements. Ideally, ion-chamber based dosimetry measurements would be more suitable. However, the irradiation field size is quite small with the current system setup and comparable to the active volume of commonly used compact parallel-plate ion chambers, such as the PTW-Advanced Markus chamber. This limitation gives rise to challenges associated with achieving lateral charge equilibrium. Furthermore, the instantaneous dose rate in our setup exceeds the dose rate for which the Advanced Markus chamber was originally evaluated for proton UHDR irradiation [33]. This raises concerns about potential ion recombination effects. Additionally, without precise beam control based on BMIC, the utility of ion chambers for dose measurements is limited. We intend to provide a comprehensive report on ion chamber dose measurements once the evaluation and integration of the transmission chamber into the UHDR beamline are completed, thereby addressing these limitations.

## 5. Conclusions

It is feasible to deliver proton pencil beams at UHDR dose rates using a clinical synchrotron-based proton therapy system. The capability of the system is limited by the maximum charge per spill and spill length. To fully harness the potential of synchrotron systems for pre-clinical proton FLASH experiments, the implementation of a transmission ion-chamber-based beam delivery and control system is imperative.

## Figures and Tables

**Table 1:** Charges per spill at 142.4 MeV.

Spill Length		10 ms	20 ms	30 ms	40 ms	50 ms
Charge per spill (nC)	Mean	1.43	2.76	3.91	4.50	4.96
	Standard deviation	0.14	0.27	0.16	0.10	0.10
Average synchrotron current (nA)		143.20	138.15	130.33	112.50	99.20

**Table 2:** Charges per spill at 228.7 MeV.

Spill Length		9 ms	15 ms	45 ms	75 ms
Charge per spill (nC)	Mean	0.49	0.73	1.93	3.08
	Standard deviation	0.07	0.03	0.17	0.28
Average synchrotron current (nA)		143.20	54.44	48.67	41.07

**Table 3:** Measured dose per spill, dose rate * and field size under different UHDR irradiation conditions.

Spot Pattern	Single Spot	2 × 2 Spots with 2.5 mm Spot Spacing	2 × 2 Spots with 5.0 mm Spot Spacing	2 × 2 Spots with 7.5 mm Spot Spacing
Measurement Depth (cm)	2	5	5	13.5
D100% (Gy, Mean ± SD)	121.8 ± 5.6	81.2 ± 3.8	36.3 ± 1.7	56.2 ± 2.6
Field size at D90% (mm)	0.6	1.1	2.3	3.7
Field size at D50% (FWHM, mm)	2.1	2.9	4.2	6.9
Dose rate (Gy/s)	2436.2	1624.1	726.3	1124.0

* D100% is defined as the dose at CAX. Dose rate is defined as averaged dose rate; it equals D100% divided by the delivery time (50 ms).

## Data Availability

Data are contained within the article.

## References

[1] Favaudon V., Caplier L., Monceau V., Pouzoulet F., Sayarath M., Fouillade C., Poupon M.-F., Brito I., Hupé P., Bourhis J. (2014). Ultrahigh dose-rate FLASH irradiation increases the differential response between normal and tumor tissue in mice. Sci. Transl. Med..

[2] Miles D., Sforza D., Wong J.W., Gabrielson K., Aziz K., Mahesh M., Coulter J.B., Siddiqui I., Tran P.T., Viswanathan A.N. (2023). FLASH Effects Induced by Orthovoltage X-rays. Int. J. Radiat. Oncol..

[3] Gao F., Yang Y., Zhu H., Wang J., Xiao D., Zhou Z., Dai T., Zhang Y., Feng G., Li J. (2022). First demonstration of the FLASH effect with ultrahigh dose rate high-energy X-rays. Radiother. Oncol..

[4] Zhu H., Xie D., Yang Y., Huang S., Gao X., Peng Y., Wang B., Wang J., Xiao D., Wu D. (2022). Radioprotective effect of X-ray abdominal FLASH irradiation: Adaptation to oxidative damage and inflammatory response may be benefiting factors. Med. Phys..

[5] Montay-Gruel P., Petersson K., Jaccard M., Boivin G., Germond J.-F., Petit B., Doenlen R., Favaudon V., Bochud F., Bailat C. (2017). Irradiation in a flash: Unique sparing of memory in mice after whole brain irradiation with dose rates above 100Gy/s. Radiother. Oncol..

[6] Simmons D.A., Lartey F.M., Schüler E., Rafat M., King G., Kim A., Ko R., Semaan S., Gonzalez S., Jenkins M. (2019). Reduced cognitive deficits after FLASH irradiation of whole mouse brain are associated with less hippocampal dendritic spine loss and neuroinflammation. Radiother. Oncol..

[7] Vozenin M.-C., De Fornel P., Petersson K., Favaudon V., Jaccard M., Germond J.-F., Petit B., Burki M., Ferrand G., Patin D. (2019). The Advantage of FLASH Radiotherapy Confirmed in Mini-pig and Cat-cancer Patients. Clin. Cancer Res..

[8] Fouillade C., Curras-Alonso S., Giuranno L., Quelennec E., Heinrich S., Bonnet-Boissinot S., Beddok A., Leboucher S., Karakurt H.U., Bohec M. (2020). FLASH Irradiation Spares Lung Progenitor Cells and Limits the Incidence of Radio-induced Senescence. Clin. Cancer Res..

[9] Levy K., Natarajan S., Wang J., Chow S., Eggold J.T., Loo P.E., Manjappa R., Melemenidis S., Lartey F.M., Schüler E. (2020). Abdominal FLASH irradiation reduces radiation-induced gastrointestinal toxicity for the treatment of ovarian cancer in mice. Sci. Rep..

[10] Montay-Gruel P., Acharya M.M., Gonçalves Jorge P., Petit B., Petridis I.G., Fuchs P., Leavitt R., Petersson K., Gondré M., Ollivier J. (2021). Hypofractionated FLASH-RT as an Effective Treatment against Glioblastoma that Reduces Neurocognitive Side Effects in Mice. Clin. Cancer Res..

[11] Rohrer Bley C., Wolf F., Gonçalves Jorge P., Grilj V., Petridis I., Petit B., Böhlen T.T., Moeckli R., Limoli C., Bourhis J. (2022). Dose- and Volume-Limiting Late Toxicity of FLASH Radiotherapy in Cats with Squamous Cell Carcinoma of the Nasal Planum and in Mini Pigs. Clin. Cancer Res..

[12] Zhang Q., Cascio E., Li C., Yang Q., Gerweck L.E., Huang P., Gottschalk B., Flanz J., Schuemann J. (2020). FLASH Investigations Using Protons: Design of Delivery System, Preclinical Setup and Confirmation of FLASH Effect with Protons in Animal Systems. Radiat. Res..

[13] Velalopoulou A., Karagounis I.V., Cramer G.M., Kim M.M., Skoufos G., Goia D., Hagan S., Verginadis I.I., Shoniyozov K., Chiango J. (2021). FLASH Proton Radiotherapy Spares Normal Epithelial and Mesenchymal Tissues While Preserving Sarcoma Response. Cancer Res..

[14] Singers Sørensen B., Krzysztof Sitarz M., Ankjærgaard C., Johansen J., Andersen C.E., Kanouta E., Overgaard C., Grau C., Poulsen P. (2022). In vivo validation and tissue sparing factor for acute damage of pencil beam scanning proton FLASH. Radiother. Oncol..

[15] Sørensen B.S., Sitarz M.K., Ankjærgaard C., Johansen J.G., Andersen C.E., Kanouta E., Grau C., Poulsen P. (2022). Pencil beam scanning proton FLASH maintains tumor control while normal tissue damage is reduced in a mouse model. Radiother. Oncol..

[16] Penn Vet|Clinical Trial Detail-Evaluation of FLASH Proton RT in Naturally Occurring Canine Head and Neck Cancer. https://www.vet.upenn.edu/research/clinical-trials-vcic/all-clinical-trials/clinical-trial/evaluation-of-flash-proton-rt-in-naturally-occurring-canine-head-and-neck-cancer.

[17] FLASH Radiotherapy for the Treatment of Symptomatic Bone Metastases in the Thorax. Clinical Trial Registration NCT05524064. March 2023. NCT05524064.

[18] Diffenderfer E.S., Verginadis I.I., Kim M.M., Shoniyozov K., Velalopoulou A., Goia D., Putt M., Hagan S., Avery S., Teo K. (2020). Design, Implementation, and in Vivo Validation of a Novel Proton FLASH Radiation Therapy System. Int. J. Radiat. Oncol..

[19] Patriarca A., Fouillade C., Auger M., Martin F., Pouzoulet F., Nauraye C., Heinrich S., Favaudon V., Meyroneinc S., Dendale R. (2018). Experimental Set-up for FLASH Proton Irradiation of Small Animals Using a Clinical System. Int. J. Radiat. Oncol..

[20] Yang Y., Kang M., Chen C.-C., Hu L., Yu F., Tsai P., Huang S., Liu J., Turner R., Shen B. (2023). Commissioning a 250 MeV research beamline for proton FLASH radiotherapy preclinical experiments. Med. Phys..

[21] Charyyev S., Chang C.-W., Zhu M., Lin L., Langen K., Dhabaan A. (2023). Characterization of 250 MeV Protons from the Varian ProBeam PBS System for FLASH Radiation Therapy. Int. J. Part. Ther..

[22] Darafsheh A., Hao Y., Zwart T., Wagner M., Catanzano D., Williamson J.F., Knutson N., Sun B., Mutic S., Zhao T. (2020). Feasibility of proton FLASH irradiation using a synchrocyclotron for preclinical studies. Med. Phys..

[23] Darafsheh A., Hao Y., Zhao X., Zwart T., Wagner M., Evans T., Reynoso F., Zhao T. (2021). Spread-out Bragg peak proton FLASH irradiation using a clinical synchrocyclotron: Proof of concept and ion chamber characterization. Med. Phys..

[24] Yang M., Wang X., Guan F., Titt U., Iga K., Jiang D., Takaoka T., Tootake S., Katayose T., Umezawa M. (2022). Adaptation and dosimetric commissioning of a synchrotron-based proton beamline for FLASH experiments. Phys. Med. Biol..

[25] Titt U., Yang M., Wang X., Iga K., Fredette N., Schueler E., Lin S.H., Zhu X.R., Sahoo N., Koong A.C. (2022). Design and validation of a synchrotron proton beam line for FLASH radiotherapy preclinical research experiments. Med. Phys..

[26] Iwata H., Toshito T., Omachi C., Umezawa M., Shinozawa Y., Yamada M., Nakajima K., Nomura K., Ogino H., Shibamoto Y. (2020). Scanning Proton FLASH Irradiation Using a Synchrotron Accelerator: Effects on Cultured Cells and Differences by Irradiation Positions. Int. J. Radiat. Oncol. Biol. Phys..

[27] Zou W., Diffenderfer E.S., Ota K., Boisseau P., Kim M.M., Cai Y., Avery S.M., Carlson D.J., Wiersma R.D., Lin A. (2021). Characterization of a high-resolution 2D transmission ion chamber for independent validation of proton pencil beam scanning of conventional and FLASH dose delivery. Med. Phys..

[28] Jaccard M., Petersson K., Buchillier T., Germond J.-F., Durán M.T., Vozenin M.-C., Bourhis J., Bochud F.O., Bailat C. (2017). High dose-per-pulse electron beam dosimetry: Usability and dose-rate independence of EBT3 Gafchromic films. Med. Phys..

[29] Guan F., Wang X., Yang M., Draeger E., Han D., Iga K., Guo F., Perles L., Li Y., Sahoo N. (2023). Dosimetric response of Gafchromic^TM^ EBT-XD film to therapeutic protons. Precis. Radiat. Oncol..

[30] Villoing D., Koumeir C., Bongrand A., Guertin A., Haddad F., Métivier V., Poirier F., Potiron V., Servagent N., Supiot S. (2022). Technical note: Proton beam dosimetry at ultra-high dose rates (FLASH): Evaluation of GAFchromic^TM^ (EBT3, EBT-XD) and OrthoChromic (OC-1) film performances. Med. Phys..

[31] Lewis D., Micke A., Yu X., Chan M.F. (2012). An efficient protocol for radiochromic film dosimetry combining calibration and measurement in a single scan. Med. Phys..

[32] Folkerts M.M., Abel E., Busold S., Perez J.R., Krishnamurthi V., Ling C.C. (2020). A framework for defining FLASH dose rate for pencil beam scanning. Med. Phys..

[33] Yin L., Zou W., Kim M.M., Avery S.M., Wiersma R.D., Teo B.-K.K., Dong L., Diffenderfer E.S. (2022). Evaluation of Two-Voltage and Three-Voltage Linear Methods for Deriving Ion Recombination Correction Factors in Proton FLASH Irradiation. IEEE Trans. Radiat. Plasma Med. Sci..

